# Improved soluble expression and use of recombinant human renalase

**DOI:** 10.1371/journal.pone.0242109

**Published:** 2020-11-12

**Authors:** Clifford S. Morrison, Elena E. Paskaleva, Marvin A. Rios, Thomas R. Beusse, Elaina M. Blair, Lucy Q. Lin, James R. Hu, Aidan H. Gorby, David R. Dodds, William B. Armiger, Jonathan S. Dordick, Mattheos A. G. Koffas

**Affiliations:** 1 Department of Chemical and Biological Engineering, Rensselaer Polytechnic Institute, Troy, New York, United States of America; 2 Center for Biotechnology and Interdisciplinary Studies, Rensselaer Polytechnic Institute, Troy, New York, United States of America; 3 Department of Chemical Engineering, Brigham Young University, Provo, Utah, United States of America; 4 Department of Biological Sciences, Rensselaer Polytechnic Institute, Troy, New York, United States of America; 5 BiochemInsights, Malvern, Pennsylvania, United States of America; 6 Department of Biomedical Engineering, Rensselaer Polytechnic Institute, Troy, New York, United States of America; University of Houston, UNITED STATES

## Abstract

Electrochemical bioreactor systems have enjoyed significant attention in the past few decades, particularly because of their applications to biobatteries, artificial photosynthetic systems, and microbial electrosynthesis. A key opportunity with electrochemical bioreactors is the ability to employ cofactor regeneration strategies critical in oxidative and reductive enzymatic and cell-based biotransformations. Electrochemical cofactor regeneration presents several advantages over other current cofactor regeneration systems, such as chemoenzymatic multi-enzyme reactions, because there is no need for a sacrificial substrate and a recycling enzyme. Additionally, process monitoring is simpler and downstream processing is less costly. However, the direct electrochemical reduction of NAD(P)^+^ on a cathode may produce adventitious side products, including isomers of NAD(P)H that can act as potent competitive inhibitors to NAD(P)H-requiring enzymes such as dehydrogenases. To overcome this limitation, we examined how nature addresses the adventitious formation of isomers of NAD(P)H. Specifically, renalases are enzymes that catalyze the oxidation of 1,2- and 1,6-NAD(P)H to NAD(P)^+^, yielding an effective recycling of unproductive NAD(P)H isomers. We designed several mutants of recombinant human renalase isoform 1 (rhRen1), expressed them in *E*. *coli* BL21(DE3) to enhance protein solubility, and evaluated the activity profiles of the renalase variants against NAD(P)H isomers. The potential for rhRen1 to be employed in engineering applications was then assessed in view of the enzyme’s stability upon immobilization. Finally, comparative modeling was performed to assess the underlying reasons for the enhanced solubility and activity of the mutant enzymes.

## Introduction

There are nearly 700 known classes of redox enzymes that use NAD(P)H as reduced cofactor [1]. Nearly 20% of known oxidoreductases require cofactors to supply stoichiometric quantities of reducing equivalents. For the majority of these enzymes, NAD(P)H is the required cofactor. In some reports, NAD(P)H is referred to, in its enzymatically active form, as 1,4-NAD(P)H in order to specifically reflect that the hydrogen on the dihydropyridyl moiety is at the C-4 position. Other reported names for non-phosphorylated 1,4-NADH include β-NADH, DPNH, and coenzyme II [2]. 1,4-NAD(P)H is significant in a variety of biocatalytic processes. For example, the enzymatic production of butanol [3] and ethanol [4]; the hydroxylation of alkanes and aromatic compounds [5]; the epoxidation of alkenes [6]; heteroatom oxygenations [7]; and Baeyer–Villiger reactions [8] are a few of the relevant biotransformations that utilize 1,4-NAD(P)H as a redox cofactor [2, 9].

1,4-NAD(P)H is consumed stoichiometrically in biocatalytic reduction reactions, but its high cost and lability [10] present an imposing economic obstacle for industrial processes without the ability to reduce NAD(P)^+^ back to 1,4-NAD(P)H. Furthermore, high concentrations of 1,4-NAD(P)H can result in competitive enzyme inhibition [11]. Therefore, there is clear motivation to achieve continuous *in situ* regeneration of 1,4-NAD(P)H from a limited pool of NAD(P)^+^ [12]. Several biocatalytic approaches to 1,4-NAD(P)H regeneration have been investigated, including whole cell and coupled enzyme/substrate methods [2, 12–14].

As opposed to enzymatic cofactor regeneration, which is highly selective for the catalytically active form 1,4-NAD(P)H, non-biological cofactor regeneration strategies are driven by nonspecific electrochemical, photochemical or chemical reduction processes [12]. Such nonspecific reactions result in a mixture of reduced nicotinamide ring isomers, including the desired 1,4-NAD(P)H species and the non-biologically active 1,2-NAD(P)H and 1,6-NAD(P)H isomers [15]. Potential strategies for mitigating the formation of [NAD(P)]_2_ in electrochemical bioreactor systems include recovery by photochemical reactions, electrochemical oxidation, and enzymatic reactions; and preventative physicochemical measures such as carefully controlling the pH of the reaction mixture and monitoring dissolved O_2_ discourage dimer formation in the first place. These and other strategies are discussed in detail by Morrison et al. [2]. The adventitious formation of these enzymatically non-productive isomers, as well as dimers of [NAD(P)]_2_, leads to inefficiency in cofactor regeneration systems and ultimately limits recycle turnover due to loss of functional cofactor.

The inhibitory effect of these isomers and other documented impurities in cofactor preparations on enzymes, such as alcohol dehydrogenase (ADH) and lactate dehydrogenase (LDH), is well-established [16–18]. Additionally, 1,6-NADH has been shown to be a potent competitive inhibitor of LDH from pig heart tissue with a K_i_ of approximately 0.6 μM [19]. A similar result was obtained with LDH from rabbit muscle with a K_i_ of 0.2 μM [20]. Both 1,6-NADH and 1,2-NADH inhibit LDH and malate dehydrogenase (MDH) [21]. *E*. *coli* MDH had high affinity for 1,6-NADH with a K_i_ of approximately 34.0 nM, while the affinity for 1,2-NADH was more modest with a K_i_ of approximately 3.0 μM. Given that these enzymes are central to primary metabolism, any accumulation of these isomers *in vivo* may present a serious concern. The problems of inefficiency in cofactor regeneration and enzyme inhibition are well-recognized as major reasons for the current inability of non-enzymatic 1,4-NAD(P)H regeneration methods to be competitive with enzymatic regeneration [15].

In 2005, Xu et al. reported the identification of a novel flavin adenine dinucleotide-dependent amine oxidase secreted by the kidneys into the blood and denoted as renalase. The group further cloned and expressed this enzyme in *E*. *coli* BL21 as recombinant human renalase isoform 1 (rhRen1). Although levels of hRen1 gene expression were found to be highest in the kidneys, hRen1 was also detected in heart and skeletal muscle tissues by Northern blot analysis. Interestingly, the primary structure of hRen1 was found to be somewhat related to monoamine oxidases and the enzyme had activity against primary amines and neurologically-relevant catecholamines. Indeed, while having just a superficial resemblance to monoamine oxidases, rhRen1 seemingly had markedly higher activity against catecholamines versus primary amines, which initially suggested that the enzyme naturally functions to regulate ambulatory blood pressure by metabolizing circulating catecholamines [22]. However, Pandini et al. demonstrated that there was no significant consumption of O_2_ by rhRen1 over the autooxidation rates of dopamine, epinephrine, norepinephrine, serotonin, benzylamine, or tyramine [23].

As a result of this apparent disparity in catalytic function, additional studies were performed to elucidate renalase function. Specifically, Beaupre et al. [21] and Hoag et al. [24] conclusively showed that rhRen1 is active against the biologically-non-productive 1,2-NAD(P)H and 1,6-NAD(P)H isomers while inactive against the biologically active 1,4-NAD(P)H isomer. The non-productive isomers of NAD(P)H are believed to arise *in vivo* through tautomerization and non-specific biochemical redox events [21]. For this reason, it is believed that hRen1 likely acts as a scavenging enzyme *in vivo* to prevent the accumulation of non-productive and inhibitory isomers of NAD(P)H within cells and to oxidize the undesired isomers back to the biochemically-useful NAD(P)^+^ state [21].

A particularly promising application for rhRen1 is in the field of electrochemical cofactor regeneration, in which an *in situ* cofactor recycling system is used to maintain a constant supply of reduced cofactor available to a biocatalytic process *in vitro*. As indicated above, continuous addition of fresh 1,4-NAD(P)H into the reaction is costly at large scale and inefficient as accumulated NAD(P) may competitively inhibit key cofactor-requiring enzymes [11]. Therefore, it is desirable to supply a minimal amount of oxidized cofactor into a biocatalytic system and reduce it as needed with an electrolytic cell. However, nonspecific electrochemical reduction of NAD(P)^+^ on an electrode surface is mediated by reactions involving free radicals, which results in the adventitious formation of many undesired side products, including non-productive NAD(P)H isomers and [NAD(P)^+^]_2_, which would quickly result in loss of biologically useful 1,4-NAD(P)^+^ [2]. Reduction of the rate of [NAD(P)^+^]_2_ formation, as well as NAD(P)H isomer formation, can be achieved by modifying the physical characteristics of electrodes, e.g. by immersing an electrode in a solution of histidine or cholesterol to form a liquid crystal membrane over the electrode surface [1, 25–32]. However, no enzymatic mitigation strategy exists to recover NAD(P)^+^ or NAD(P)H from non-productive and inhibitory NAD(P)H isomers.

To this end, rhRen1 may provide a functional solution for cofactor recycling in electrochemical reduction reactions [33]. However, rhRen1 is a difficult protein to express in *E*. *coli* without formation of inclusion bodies. Pandini et al. investigated a variety of expression strains and culture conditions on the soluble expression of rhRen1 [23]. The various expression strains tested in their work, induced with isopropyl β-D-1-thiogalactopyranoside (IPTG), had minimal advantage over the use of a conventional *E*. *coli* BL21 (DE3) expression strain and that immunochemically-detectable titers of rhRen1 were only found when the temperature of the fermentation was below 20°C. Co-expression of *E*. *coli* chaperone proteins did not improve the yield of soluble protein [23]. Desir et al. reported some success in purifying rhRen1 from inclusion bodies and refolding the protein in the presence of FAD, albeit the process is rigorous and unlikely to be scalable [34]. It is also unclear whether the refolded protein retains inherent catalytic activity following refolding. As a result, the highest reported production titer for rhRen1 is approximately 6 mg L^-1^ of culture [35]. Based on this limitation, we pursued a protein engineering strategy to produce highly active and soluble rhRen1 mutants at high yield. This work provides a foundation for using a biocatalytic solution to address the key drawback of electrochemical NAD(P)^+^ reduction reactions for a highly diverse array of biotransformations.

## Materials and methods

### Improved solubility

The genes for wild type hRen1 (WThRen1), m5hRen1, and m6hRen1, codon optimized for expression in *E*. *coli*, were cloned into a pET-32b(+) vector using the *Nde*I closest to the ribosome binding site and *Xho*I restriction sites. The genes for WThRen1-SUMO, m5hRen1-SUMO, m6hRen1-SUMO, and WThRen1-MBP, m5hRen1-MBP, and m6hRen1-MBP, all codon optimized for expression in *E*. *coli*, were cloned into a pET-His6-SUMO-TEV-LIC (2ST) vector by *Ssp*I blunt end ligation. All genes were supplied by GenScript USA. The full maps for each of these constructs is provided in S1-S9 Figs in S1 File.

The vectors include a T7 promoter for induction by isopropyl β-D-1-thiogalactopyranoside (IPTG), an ampicillin resistance gene for selection pressure, and a 6xHis tag for purification on a Ni^2+^-nitrilotriacetic acid (NTA) agarose resin. Each construct was transformed into *E*. *coli* BL21(DE3) and plated on agar containing ampicillin (80 μg mL^-1^). Colonies were selected from the plate and grown into overnight cultures. LB medium (50 mL), also containing ampicillin (80 μg mL^-1^), was inoculated at a ratio of 1% from the overnight culture and allowed to grow at 37°C until OD_600_ = 0.5. At this point, select batches were induced with 0.1 mM IPTG and the temperature of all batches were reduced to 22°C and allowed to express protein for up to 22 h.

After approximately 22 h of protein expression, the cells were collected, pelleted, washed, and resuspended in phosphate buffered saline (PBS) buffer (pH, 7.4). Cell lysis was achieved by sonication on ice at 30% amplitude in 1 s pulses for 5 min. The cell lysate was then centrifuged again to separate the soluble fraction from the insoluble fraction. Each sample was clarified by repeated centrifugation and short passes on an Ni-NTA column, normalized by culture volume with PBS, and analyzed by SDS-PAGE to demonstrate the relative proportions of enzyme production per plasmid construct.

### Wild-type and mutant renalase kinetics and stability

NAD^+^ at a concentration of 19.33 mM was chemically reduced with excess NaBH_4_ to produce a vividly yellow 1:1:1 stoichiometric equilibrium mixture of each NADH isomer. This equilibrium mixture was then taken as the stock substrate for subsequent renalase kinetics assessments, and the amount of substrate added to the reaction mixture is reported here in terms of volume added to the renalase reaction mixture. The UV-Vis λ_max_ of each isomer was chosen as the wavelength of analysis for a negative control kinetic experiment, i.e., the reaction mixture containing only PBS and a sample of the substrate mixture.

Due to the inherent instability of the 1,2-NADH isomer, reactions were performed on a time scale of minutes. For subsequent analyses, 420 nm was chosen instead of 395 nm to eliminate the contribution of 1,4-NADH and 1,6-NADH to the signal, thereby providing a direct measurement of the concentration of 1,2-NADH in the equilibrium isomer mixture. The molar absorptivity absorption spectra for the isomers is given in the work by Beaupre et al. [21] and was also provided directly by the authors.

Each enzyme variant was purified using Ni-NTA resin, extensively washing the loaded resin with PBS, and eluting the protein with 500 mM imidazole to give a sample with a bright yellow color due to the FAD docked within the enzyme. Imidazole in the resulting samples was removed by exchanging with PBS by ultrafiltration. Finally, glycerol was added to the enzyme samples at a concentration of 20% as a cryoprotectant for storage at −80°C. Aliquots of the frozen enzyme samples were then taken for all subsequent analyses.

Specific activity was assessed by holding the concentration of the substrate mixture constant and varying the amount of enzyme in each reaction, then measuring the rate of change of optical density at 420 nm (ΔmOD_420_) as a function of the amount of enzyme present in the reaction in replicate. The slope of the line resulting from plotting the average values of ΔmOD_420_ min^-1^ gives the specific activity of each enzyme.

### Enzyme immobilization

Ni-NTA resin (2 mL) charged with 0.011 mg of m5hRen1 per mL of resin in a 10 mL reaction volume for a total of 0.0022 mg of enzyme present in the reaction. At 3 min intervals, the reaction was stopped by filtration, and replicate samples of the resultant reaction mixture were analyzed to provide a piece-wise kinetic profile of the enzyme activity over time. The slope of the line given by plotting ΔmOD_420_ vs. time provides the velocity of the enzyme in units of ΔmOD_420_ min^-1^.

### Comparative modeling of renalase mutants

The crystal structure of WThRen1 docked with FAD is available in the PDB database as PDB ID: 3QJ4. Homology modeling was used to generate 3D models of the renalase mutants based on the solved crystal structure of WThRen1. WThRen1 was mutagenized manually in PyMOL to generate a PDB file with the correct primary sequences for m5hRen1 and m6hRen1. However, these mutagenized models do not necessarily exhibit the correct side chain conformations. The SCWRL4 algorithm was then implemented on the mutagenized renalase models to correct the rotamer orientations [36]. Finally, molecular graphics and analyses performed with UCSF Chimera, developed by the Resource for Biocomputing, Visualization, and Informatics at the University of California, San Francisco, were performed for energy minimization of the rotamer-corrected 3D structures [37]. The 3D models of WThRen1, m5hRen1, and m6hRen1 were modeled in PyMOL and aligned to provide direct comparison.

## Results and discussion

### Improved solubility

To address the poor solubility of rhRen1 in *E*. *coli*, two parallel strategies were pursued; engineering of the protein’s primary sequence and use of soluble fusion proteins. The former was addressed initially using the PROSS (Protein Repair One-Stop Shop) algorithm and based on the wild-type WThRen1 3D structure. The PROSS algorithm proposes mutations of the input protein that are expected to address issues related to poor solubility, low expression levels, misfolding, aggregation, and other stability factors [38]. Seven possible mutants were proposed, each with increasing numbers of mutations to the WT sequence. Designs #5 and #6 (henceforth referred to as mutation set m5 and mutation set m6) were chosen for analysis relative to the WT enzyme. m5 and m6 were chosen on the basis of cost-effectiveness. Previous experience with PROSS-generated mutants suggested that candidates with few mutations sometimes do not provide much stabilization or solubilization while candidates with the most mutations sometimes sacrifice reactivity. Further exploration of the untested candidates may provide additional biochemical insights into better solubility and/or reactivity of mutant renalase. The primary sequences of these proteins and a multiple sequence alignment performed by the Clustal Omega program are given in S1 Table in S1 File. m5 introduced 19 mutations and m6 introduced 27 mutations to the primary sequence of WT renalase. Both m5 and m6 included one replacement for a cysteine residue to the WT sequence. Plasmid construct maps are given in S1-S9 Figs in S1 File.

Next, protein fusion tags known to enhance solubility in *E*. *coli* were considered. A small ubiquitin-like modifier (SUMO) tag had previously been explored by Pandini et al. [23] and resulted mostly in the enhanced expression of the protein in the insoluble fraction of the cell lysate, providing effectively no advantage over the soluble expression of the non-fused enzyme. The authors did not explore other fusion proteins. Based on the enhanced, albeit insoluble, expression of WThRen1, the use of a SUMO tag was chosen herein as an initial protein fusion candidate. An MBP tag was also chosen as a common method for improved protein solubility [39]. Altogether, the three rhRen1 variants (WT, m5, m6) and the chimeric variants (SUMO fusion, MBP fusion for each of WT, m5 and m6) formed a small library of nine renalase constructs for solubility and activity evaluations. The theoretical pI and molecular weights of the nine protein variants as predicted by PyMOL are given in S2 Table in S1 File.

SDS-PAGE was performed following expression of the nine protein variants. SDS-PAGE analysis of the soluble fraction of the cell lysate clearly indicates that the m5hRen1 and m6hRen1 variants are dramatically more soluble than WThRen1 (S10 Fig in S1 File). The production titers of m5Ren1 and m6hRen1 were found to be approximately 50 mg L^-1^ and 120 mg L^-1^, respectively, suggesting as much as a 20-fold improvement in solubility over previous studies. There is also agreement with the results from Pandini et al. [23] regarding the inability of a fusion tag alone to impart solubility to WThRen1. It should also be noted that there was some observed leakage in expression of rhRen1 even in the absence of IPTG. It is interesting to note that the highest overall protein expression came from the WThRen1-MBP construct. However, all of the protein was expressed in the insoluble fraction (S11-S12 Figs in S1 File). This suggests that while MBP was clearly beneficial for helping to express the protein, it did not enhance WT enzyme solubility.

The plasmid construct for m6hRen1-SUMO was arbitrarily selected to assess the effect of temperature on the batch fermentation process. Three different temperatures (22°C, 25°C, 30°C) were assessed at five time points, and the samples were concentrated by 10X and analyzed by SDS-PAGE. There was no advantage to performing the batch fermentation at higher temperatures, nor was there an advantage to extending the expression time beyond 16 h (S13 Fig in S1 File). Given the propensity of renalase to aggregate, a lower temperature was chosen to ensure reduced rate of incorrect folding and incorporation into insoluble inclusion bodies.

### Wild-type and mutant renalase kinetics and stability

**Fig 1:**
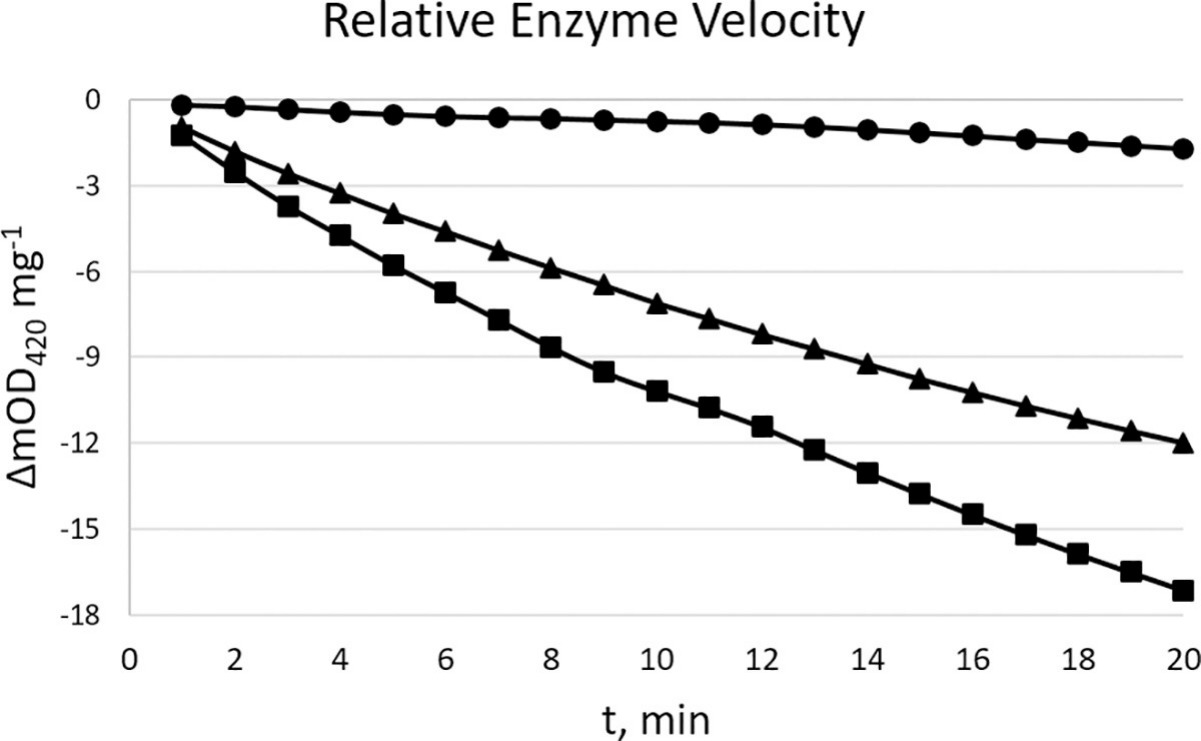
Data from a single representative experiment in which [1,2-NADH] = 1.21 mM in the equilibrium mixture of isomers, [WThRen1] = 0.72 mg mL^-1^ (filled circles) [m5hRen1] = 0.17 mg mL^-1^, (filled squares), and [m6hRen1] = 0.27 mg mL^-1^ (filled triangles).

To assess the kinetic parameters of the chimeric renalase mutants, an assay was developed based on Beaupre et al. [21]. Only two enzyme variants, m5hRen1 and m6hRen1, provided both enhanced solubility (S10 Fig in S1 File) and enhanced activity (S14-S22 Figs in S1 File) relative to WThRen1. This information is summarized in Table 1. Therefore, all subsequent kinetic analyses were performed with these two variants. No variant with a solubility tag had higher specific activity vs. any variant without a solubility tag. Additionally, the specific activity of each variant decreased with increasing molecular weight. These results indicate that a solubility tag has a negative impact on renalase activity, and that mutant enzymes with no fusion tag are preferable. The kinetics of the selected chimeric mutants were also assessed by measuring the change in absorbance at 420 nm (ΔmOD_420_). Both m5hRen1 and m6hRen1 were more active than the WT enzyme (Fig 1).

**Table 1:** Summary of the specific activities of the renalase variants and a qualitative assessment of their enhancement relative to WThRen1.

Enzyme Variant	Specific Activity, ΔmOD_420_ min^-1^ mg^-1^	Enhanced Specific Activity	Enhanced Solubility
WThRen1	87.6		
WThRen1-SUMO	63.0		
WThRen1-MBP	19.8		
m5hRen1	1447	+ + +	+ + +
m5hRen1-SUMO	289	+ +	+ + +
m5hRen1-MBP	91.0	+	+ + +
m6hRen1	760	+ + +	+ + +
m6hRen1-SUMO	152	+ +	+ + +
m6hRen1-MBP	111	+	+ + +

**Fig 2:**
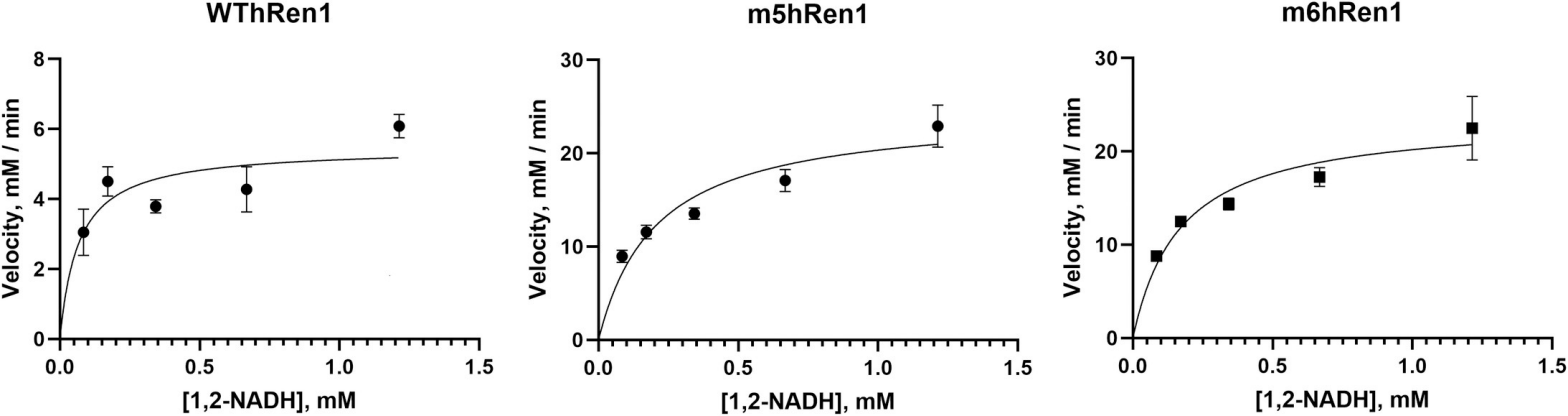
Michaelis-Menten plots for kinetic assessment of WThRen1, m5hRen1, and m6hRen1 using 1,2-NADH within an equilibrium mixture of reduced isomers as the substrate.

Michaelis-Menten kinetic parameters for 1,2-NADH were determined using non-linear regression (Fig 2). Values of V_max_ for WThRen1, m5hRen1, and m6hRen1 are 5.47 mM min^-1^, 24.5 mM min^-1^, and 23.6 mM min^-1^, respectively. The values of K_M_ are 0.07 mM, 0.21 mM, and 0.18 mM, respectively. Thus, the mutant enzymes had higher catalytic efficiencies (V_max_/K_m_ of 117 and 131 min^-1^ for m5hRen1 and m6hRen1, respectively) than WThRen1 (78 min^-1^) in addition to over 4-fold higher catalytic turnover (as reflected in V_max_).

**Fig 3:**
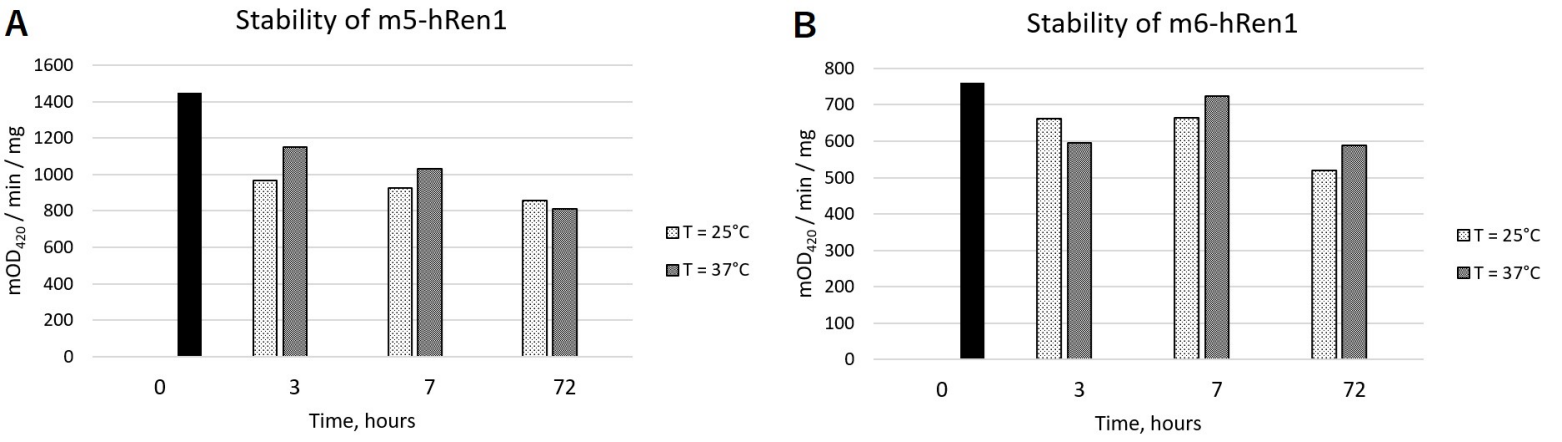
Stabilities of (A) m5hRen1 and (B) m6hRen1 as determined by measuring the specific activity of the enzyme over time at two different temperatures. The baseline specific activity is given by the black bars. Specific activity measurements at 25°C are given in the light thatched bars, and the measurements for the specific activity at 37°C are given in the dark thatched bars. [1,2-NADH] = 1.21 mM in the equilibrium mixture of isomers, [m5hRen1] = 0.17 mg mL^-1^, and [m6hRen1] = 0.27 mg mL^-1^.

Finally, the stability of m5hRen1 and m6hRen1 was evaluated by incubating samples of the enzyme at various temperatures over time and measuring the loss in specific activity of the enzyme (Fig 3). For both m5hRen1 and m6hRen1, there is some degree of activity loss over 72 h incubation. However, the degree of activity loss appears to be temperature independent. Visual inspection of the samples taken at 37°C, and to a lesser extent at 25°C, revealed that the solutions were somewhat turbid, indicating some degree of aggregation. Importantly, both mutant enzymes retained anywhere from approx. 50–80% activity after 72 h at 37°C.

### Enzyme immobilization

The results of the comparative kinetics assessments indicate that m5hRen1 is the better-performing of the renalase variants and therefore may be the preferred variant for application in NAD(P)^+^ regeneration. Immobilization of m5hRen1 to Ni-NTA and subsequent activity analysis provides insight into the effect of immobilization of m5hRen1 activity. The velocity of the reaction catalyzed by immobilized m5hRen1 under this particular set of reaction conditions is 1211 ΔmOD_420_ min^-1^ mg^-1^ of enzyme with a standard error of 70 ΔmOD_420_ min^-1^ mg^-1^ of enzyme from duplicate experiments. Under identical conditions, the velocity of free m5hRen1 was found to be 3267 ΔmOD_420_ min^-1^ mg^-1^ with a standard deviation of 589 ΔmOD_420_ min^-1^ mg^-1^ of enzyme from duplicate experiments. This indicates that there is ~ 2-3-fold loss of reaction velocity upon immobilization on Ni-NTA. Due to the low solubility of WThRen1, it was not used in immobilization experiments due to the relatively large amount of enzyme material that is required for these experiments.

### Comparative modeling of renalase mutants

**Fig 4:**
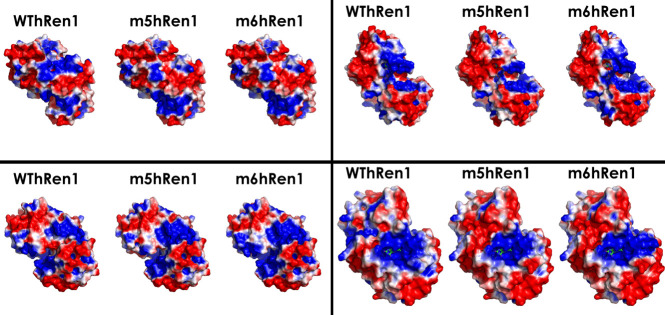
Electrostatic potential map of renalase and renalase variants. Each quadrant represents an identical perspective of a particular region of the enzyme across each variant to highlight the differences in electrostatic potential between them. Red represents regions of electronegativity, and blue represents regions of electropositivity.

To provide reasonable explanations for the aforementioned results, 3D molecular models for m5hRen1 and m6hRen1 were generated following alignment with the known crystal structure of WThRen1. The predicted pIs for these variants are not significantly different from that of the WThRen1, and this suggests that there is no major shift in polarity that is responsible for the observed solubility enhancement. Electrostatic potential surfaces, as well as hydrophobicities, were computed for WThRen1, m5hRen1, and m6hRen1 in PyMOL to explore this further. Several electrostatic regions of the enzyme have been significantly rearranged in the two mutants relative to WThRen1 (Fig 4). This rearrangement of charged moieties on the solvent-accessible surface of the enzyme is likely to be a major factor in solubility enhancement.

**Fig 5:**
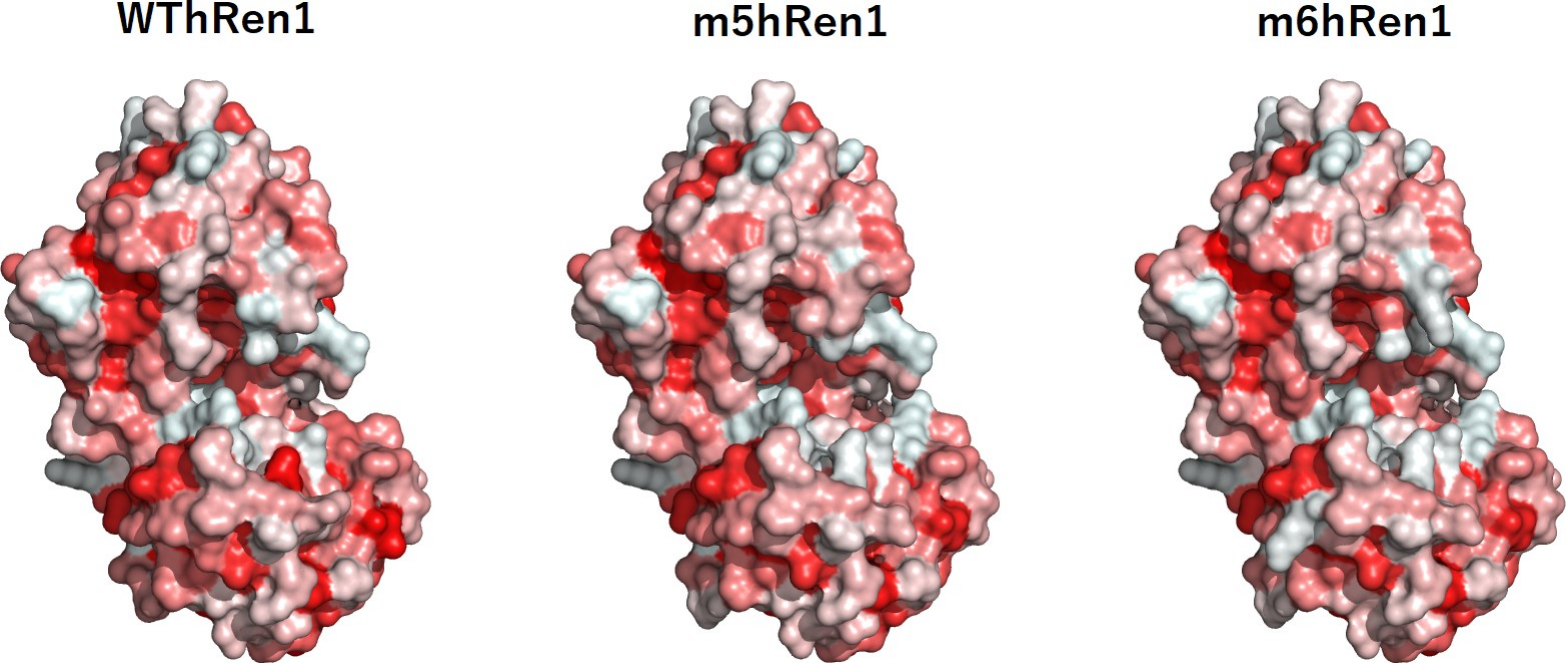
Surface hydrophobicity map of renalase and renalase variants. Some small regions of the enzyme have been significantly changed relative to WThRen1.

The predicted surface hydrophobicity for each variant was also computed (Fig 5). Although there are some small regions of hydrophobicity on the surface of the enzyme that were changed significantly relative to WThRen1, it is not likely that this contributes as much to the observed enhancement in solubility as the rearrangement of surface electrostatics.

Regions on the surface of the enzyme that were significantly changed as a result of the PROSS mutations were then analyzed. Mutations found in these specific regions of the renalase variants include Q3R, C47R, R75S, S217D, and T329Q. Each of these mutations are predicted to contribute to changes in polarity, charge, and hydrophilicity. The solvent-accessible surface area of WThRen1, as computed by PyMOL, is 17499.45 Å^2^. The solvent-accessible surface areas of the m5hRen1 and m6hRen1 models were calculated to be 17521.04 Å^2^ and 17619.59 Å^2^, respectively. These represent less than a 1% increase in the solvent-accessible surface area relative to WThRen1, so it is unlikely that an increased surface area was a major factor in the observed solubility enhancement. However, taken together with the changes to surface electrostatic potentials, surface hydrophobicity, and polarity, the sum of the contributions of these changes may explain the observed enhancement in solubility.

**Fig 6:**
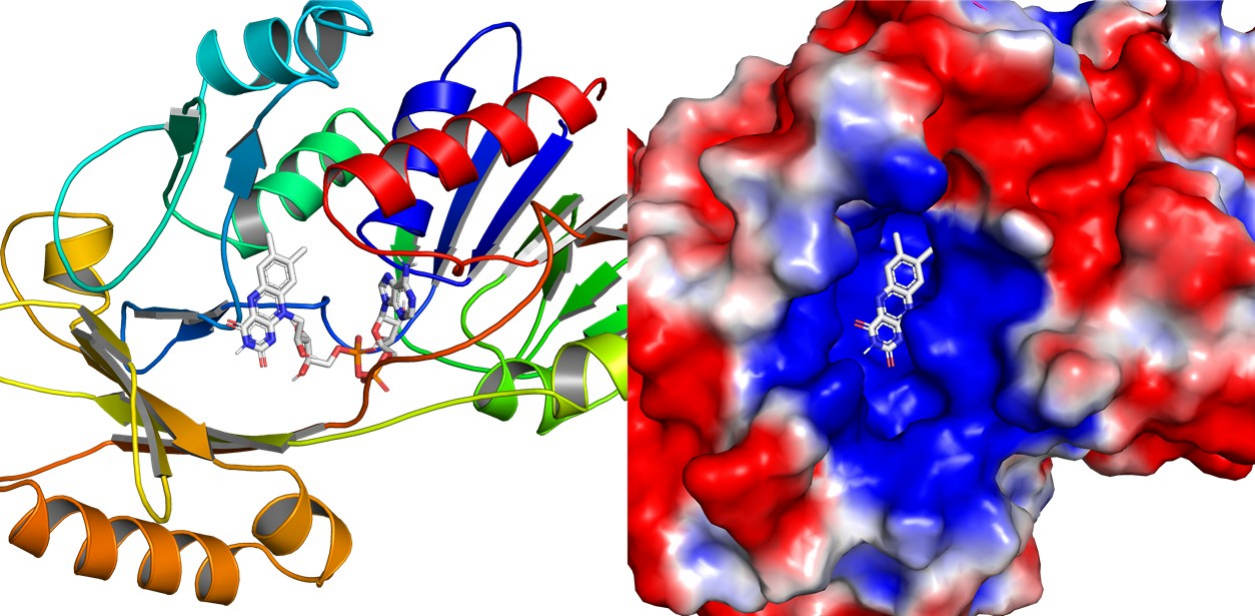
The active site of WThRen1 is marked by a localized pocket of positively-charged residues which provide access to docked FAD from the exterior of the enzyme.

To explain the enhancement in catalytic activity relative to WThRen1, the surface electrostatics of the active site were considered. The catalytic binding pocket of WThRen1 is shown in Fig 6. FAD is docked within the active site and is the source of the enzyme’s oxidation activity. As explored by Moran [40], electrons are passed from 1,2-NADH and 1,6-NADH to FAD, which then subsequently passes the electrons to water as the final electron acceptor. 1,2-NADH and 1,6-NADH are negatively-charged species, which may facilitate electron transfer to FAD.

**Fig 7:**
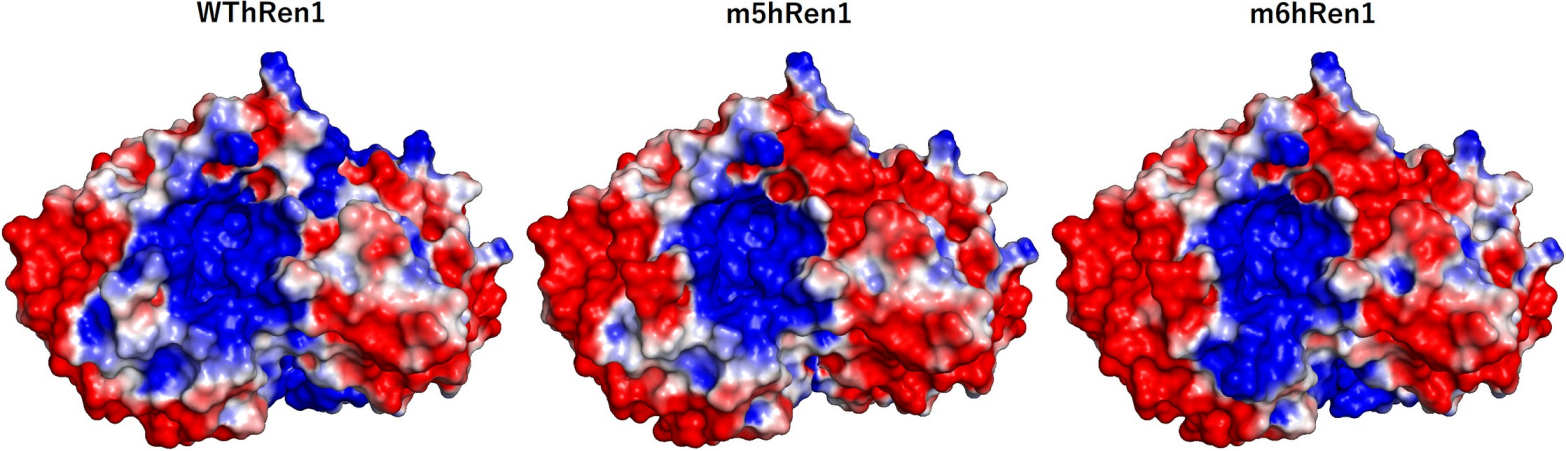
Surface electrostatic potentials of the catalytic binding pockets of the renalase variants relative to WThRen1.

Relative to WThRen1, m6hRen1 exhibits a greater density of positively-charged residues around the active site (Fig 7). This should serve to better direct the NAD(P)H isomers into the binding pocket, thereby enhancing activity. Compared to either WThRen1 or m6hRen1, m5hRen1 exhibits the greatest localization of positively-charged residues around the binding pocket.

## Conclusions

Consistent with the literature [23], fusing WThRen1 with a SUMO fusion tag resulted in a large increase in protein expression, but almost all of it was found in the insoluble fraction of the cell lysate. Since we are unaware of any previous attempts to fuse any form of WThRen1 with an MBP fusion tag, we also attempted this approach to improve rhRen1 soluble expression. Interestingly, there was a dramatic increase in overall rhRen1 expression when an MBP fusion tag was added to it, but again, almost all of it was found in the insoluble fraction of the cell lysate.

Relative to the highest protein expression levels reported in the literature, we have significantly increased the soluble expression levels of rhRen1 in *E*. *coli* BL21(DE3) by inducing mutations using the PROSS algorithm. However, even higher soluble expression levels were found when mutant rhRen1 was fused with either a SUMO fusion tag or an MBP fusion tag. Despite this, the specific activity of these variants with a solubility tag was lower than those without a solubility tag.

The mutations to the primary sequence of WThRen1 in this work largely resulted in proteins that include more hydrophilic and polar residues. Additionally, the solvent-accessible surface areas of the mutants are each marginally larger than that of the wild type. These factors, when taken together, may explain the improved soluble expression of rhRen1. Each of the proteins, WThRen1, m5hRen1 and m6hRen1, were modeled comparatively in PyMOL to identify particular mutant residues that greatly contributed to a rearranged surface electronegativity on the mutant proteins relative to the wild type. Based on the modeling performed in this work, the mutant residues in common between m5hRen1 and m6hRen1 that are thought to contribute to decreased surface electronegativity were found to be Q3R, C47R, R75S, S217D, and T329Q. It’s possible that these mutations are partly responsible for the observed solubility enhancement of the enzyme, but further analysis of the contributions from single mutations are required to validate this claim.

Taken together, these mutations served to enhance both the solubility and catalytic activity of the enzyme. In the best case, there was a ~16-fold enhancement in the specific activity of the enzyme and nearly a 2-fold increase in the catalytic efficiency. In addition, it was found that m5hRen1 can be successfully immobilized and retain activity, which may prove useful in biotransformations involving electrochemical reductions where preventing the accumulation of non-productive isomers of NAD(P)H is critical.

## Supporting information

S1 File (DOCX)

S2 File Raw images of all gels. (PDF)
