# Supplementary material for: Improved soluble expression and use of recombinant human renalase
Source: PLoS One. 2020 Nov 12;15(11):e0242109. doi: 10.1371/journal.pone.0242109 (PMC7660482; doi:10.1371/journal.pone.0242109)
Supplement: S1 File — (DOCX) [file pone.0242109.s001.docx]

**S1 Table.** **Multiple sequence alignment of WThRen1, m5hRen1, and m6hRen1 as performed by the Clustal Omega program on UniProt.**

| CLUSTAL O(1.2.4) multiple sequence alignment  WThRen1 MAQVLIVGAGMTGSLCAALLRRQTSGPLYLAVWDKAEDSGGRMTTACSPHNPQCTADLGA 60  m5hRen1 MARVLIVGAGLTGSLCAALLRRQTSGPLYLAVWDKADDSGGRMTTARSPHNPQCTADLGA 60  m6hRen1 MARVLIVGAGLTGALCAALLRRQTSGPLYLAVWDKADDSGGRMTTARSPHNPQCTADLGA 60  **:*******:**:**********************:********* *************  WThRen1 QYITCTPHYAKKHQRFYDELLAYGVLRPLSSPIEGMVMKEGDCNFVAPQGISSIIKHYLK 120  m5hRen1 QYITCTPHYAKKHQSFYDELLAHGVLRPLTSPIEGMVMKEGDCNFVAPQGISSIIKHYLK 120  m6hRen1 QYITCTPHYAKKHQSFYDELLAHGVLRPLTSPIEGMVMKEGDCNFVAPQGISSIIKHYLK 120  ************** *******:******:******************************  WThRen1 ESGAEVYFRHRVTQINLRDDKWEVSKQTGSPEQFDLIVLTMPVPEILQLQGDITTLISEC 180  m5hRen1 ESGAEVYFRHHVTQINLRDDKWEVQRETGSPEQFDLIVLTMPVPEILQLQGDITNLISEC 180  m6hRen1 ESGAEVYFRHCVTQINLRDDKWEVQRKTGSPEQFDIIILTMPVPEILQLQGDITNLISEC 180  ********** *************.::********:*:****************.*****  WThRen1 QRQQLEAVSYSSRYALGLFYEAGTKIDVPWAGQYITSNPCIRFVSIDNKKRNIESSEIGP 240  m5hRen1 QRQQLEAVSYSSRYALGLFYEAGTKIDVPWAGQYITDNPCIRFVSIDNKKRNIESSEIGP 240  m6hRen1 QRQQLEAVSYSSRYALGLFYEAGTKIDVPWAGQYITDNPCIRFISIDNKKRNIESSEIGP 240  ************************************.******:****************  WThRen1 SLVIHTTVPFGVTYLEHSIEDVQELVFQQLENILPGLPQPIATKCQKWRHSQVTNAAANC 300  m5hRen1 SLVVHTTVPFGVTHLEHSKEDVQELIFQQLENILPGLPQPVATKCQKWRHSQVTNAAANC 300  m6hRen1 SLVVHTTVPFGVKHLEHSKEDVQELIFQELENILPGLPQPVATKCQKWRYSQVTNAATNC 300  ***:********.:**** ******:**:***********:********:*******:**  WThRen1 PGQMTLHHKPFLACGGDGFTQSNFDGCITSALCVLEALKNYI 342  m5hRen1 PGQMTLHHKPFLVCGGDGFTQSNFDGCIESALCVLEALKNYI 342  m6hRen1 PGQMTLHHKPFLVCGGDGFTQSNFDGCIESALCVLEALKNYI 342  ************.*************** ************* |
| --- |

**S2 Table.** **A summary of the theoretical pI and molecular weights of the nine protein variants as predicted by PyMOL.**

| **Enzyme Variant** | **Predicted pI** | **Molecular Weight, Da** |
| --- | --- | --- |
| WThRen1 | 6.35 | 38670.15 |
| m5hRen1 | 6.25 | 38775.13 |
| m6hRen1 | 6.39 | 38836.36 |
| WThRen1-SUMO | 5.77 | 51544.56 |
| m5hRen1-SUMO | 5.77 | 51649.54 |
| m6hRen1-SUMO | 5.82 | 51710.77 |
| WThRen1-MBP | 5.63 | 80504.57 |
| m5hRen1-MBP | 5.64 | 80609.56 |
| m6hRen1-MBP | 5.68 | 80670.78 |

The genes for WThRen1, m5hRen1, and m6hRen1, codon optimized for expression in *E. coli*, were cloned into a pET-32b(+) vector using the *Nde*I (closest to the ribosome binding site) and *Xho*I restriction sites. The full plasmid maps for these constructs are shown in S1-S3 Figs.


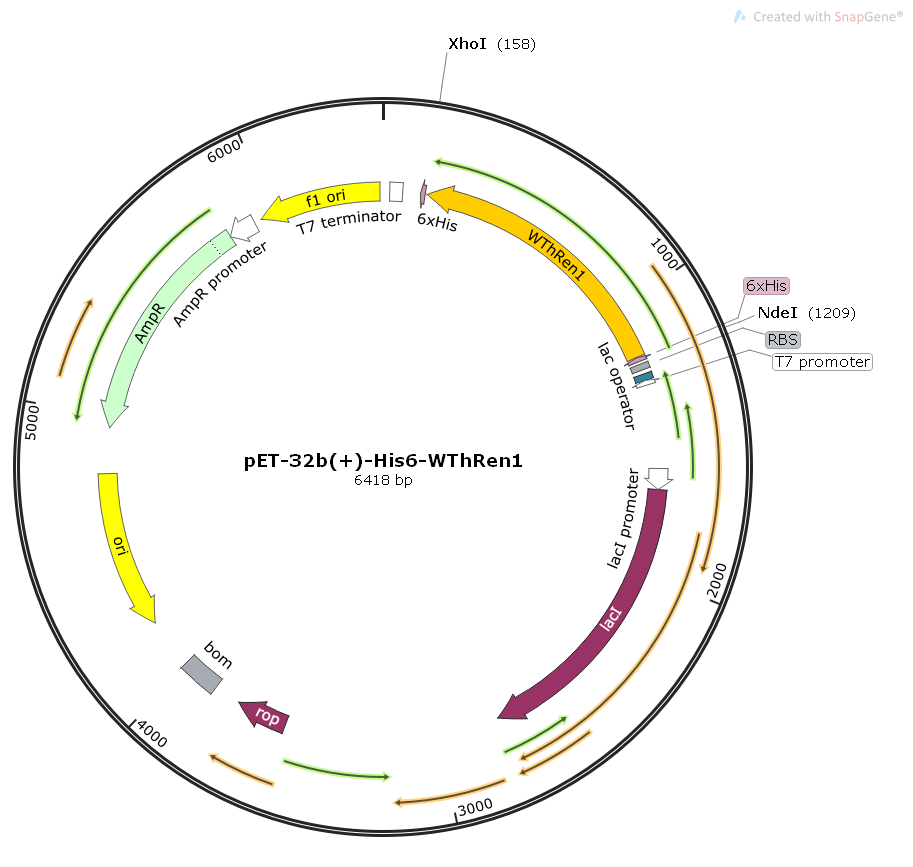


**S1 Fig.** **The full plasmid map for the construct pET-32b(+)-His6-WThRen1.**


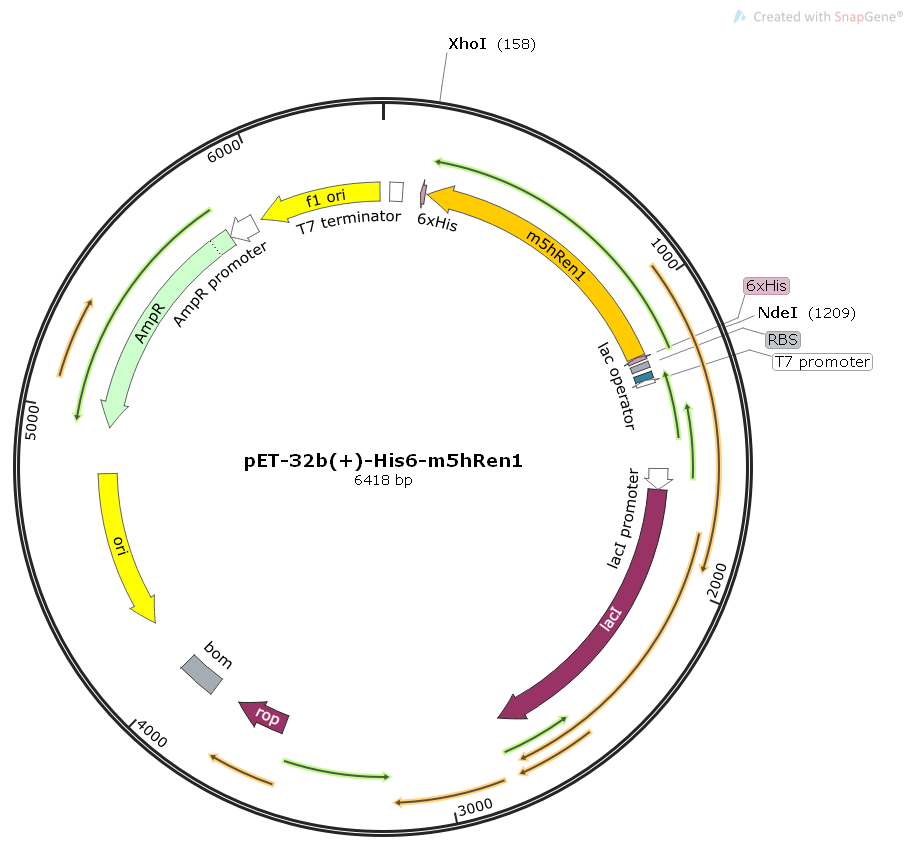


**S2 Fig.** **The full plasmid map for the construct pET-32b(+)-His6-m5hRen1.**


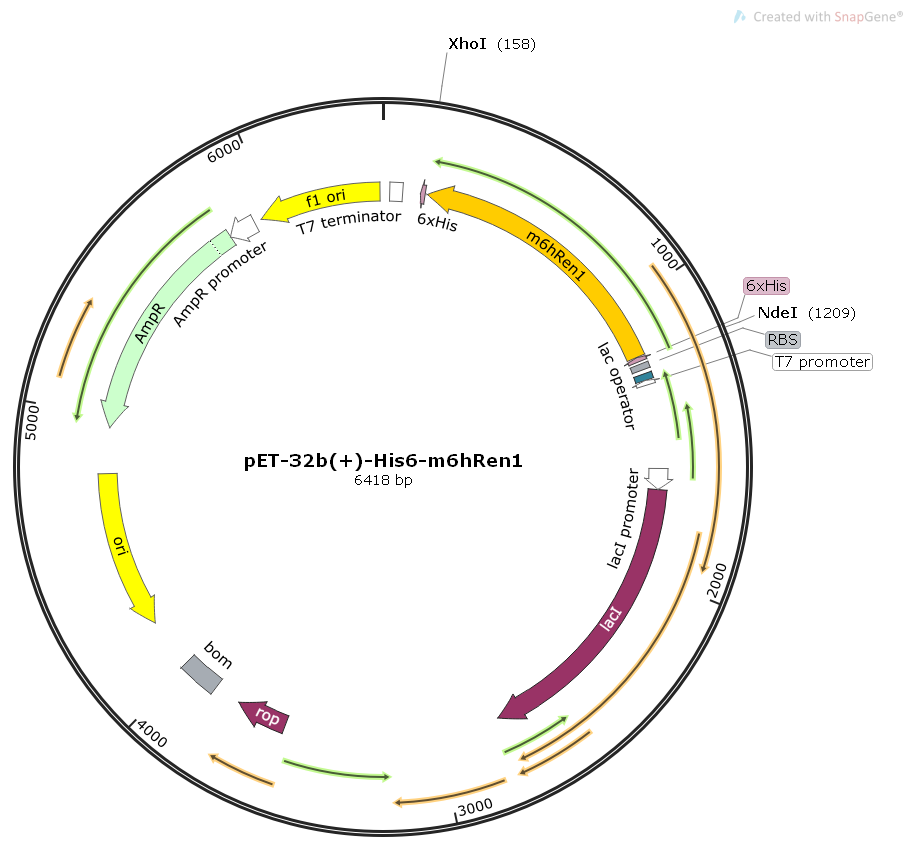


**S3 Fig.** **The full plasmid map for the construct pET-32b(+)-His6-m6hRen1.**

The genes for WThRen1-SUMO, m5hRen1-SUMO, and m6hRen1-SUMO, codon optimized for expression in *E. coli*, were cloned into a pET-His6-SUMO-TEV-LIC (2ST) vector by *Ssp*I blunt end ligation. The full plasmid maps for these constructs are shown in S4-S6 Figs.


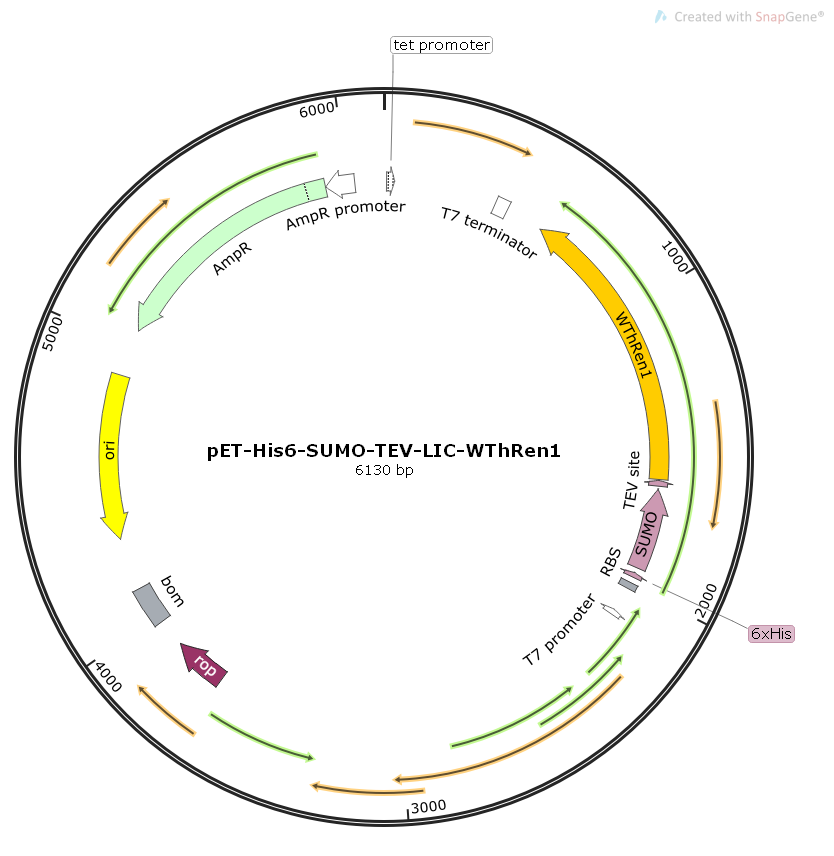


**S4 Fig.** **The full plasmid map for the construct pET-His6-SUMO-TEV-LIC-WThRen1.**


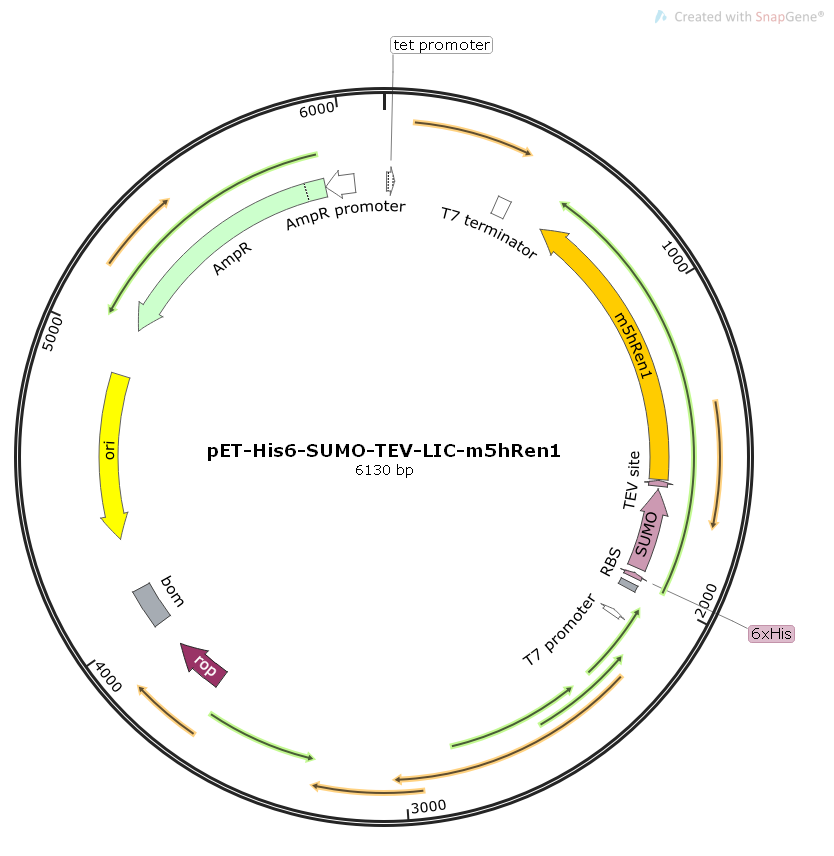


**S5 Fig. The full plasmid map for the construct pET-His6-SUMO-TEV-LIC-m5hRen1.**


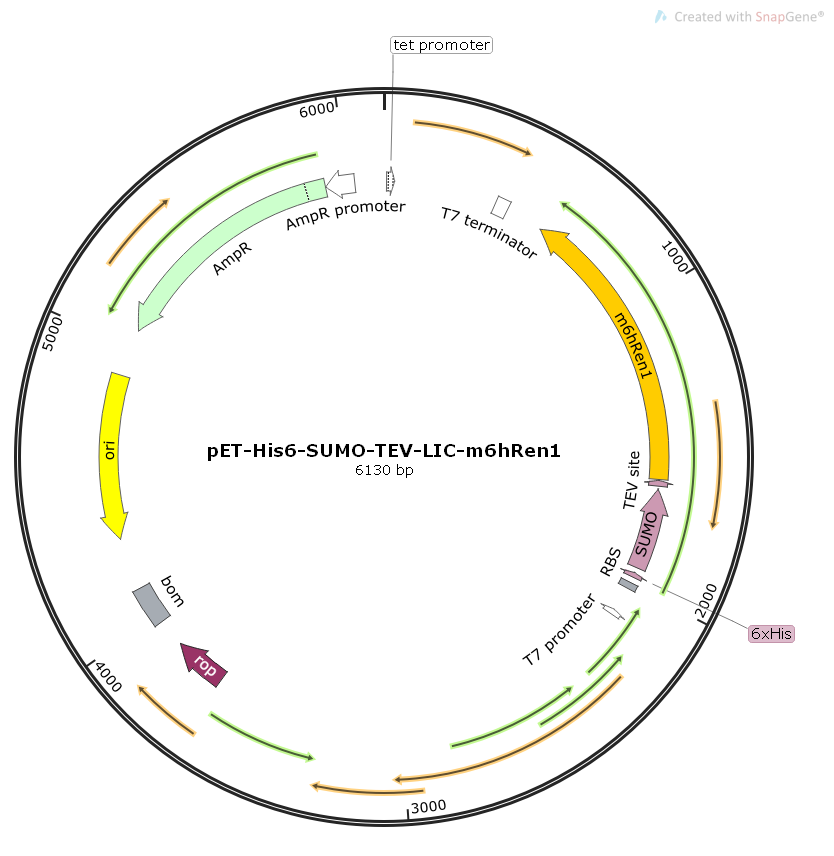


**S6 Fig.** **The full plasmid map for the construct pET-His6-SUMO-TEV-LIC-m6hRen1.**

The genes for WThRen1-MBP, m5hRen1-MBP, and m6hRen1-MBP, codon optimized for expression in *E. coli*, were cloned into a pET-His6-MBP-TEV-LIC (2ST) vector by *Ssp*I blunt end ligation. The full plasmid maps for these constructs are shown in S7-S9 Figs.


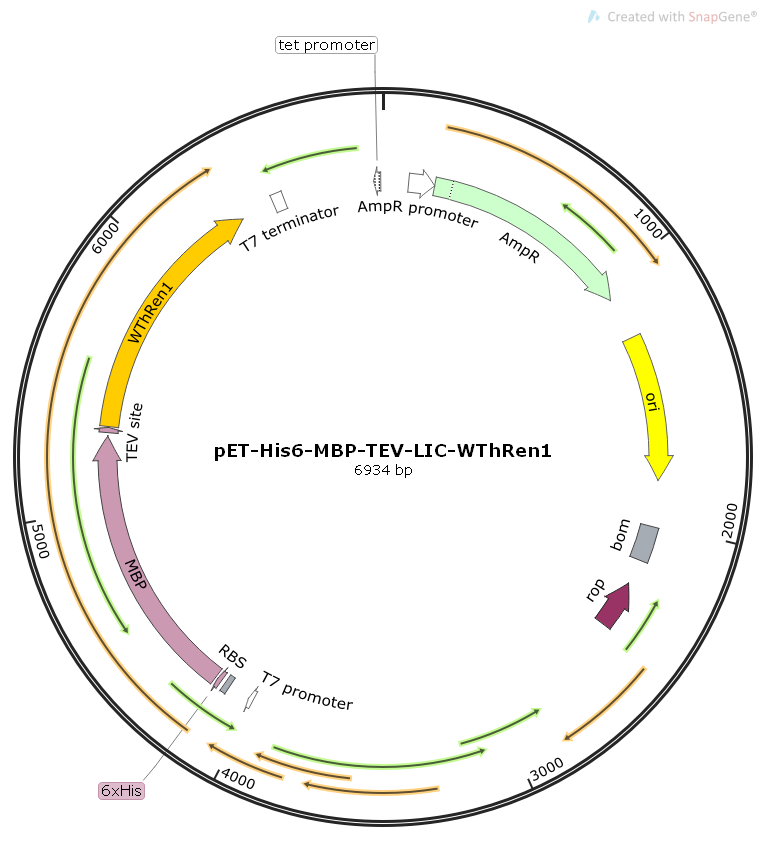


**S7 Fig.** **The full plasmid map for the construct pET-His6-MBP-TEV-LIC-WThRen1.**


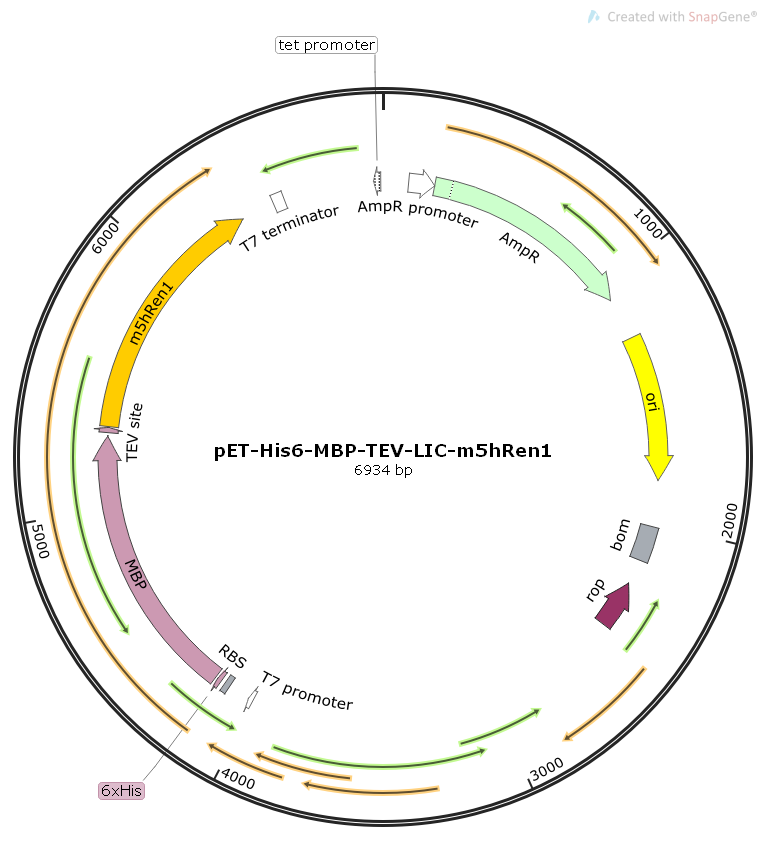


**S8 Fig. The full plasmid map for the construct pET-His6-MBP-TEV-LIC-m5hRen1.**


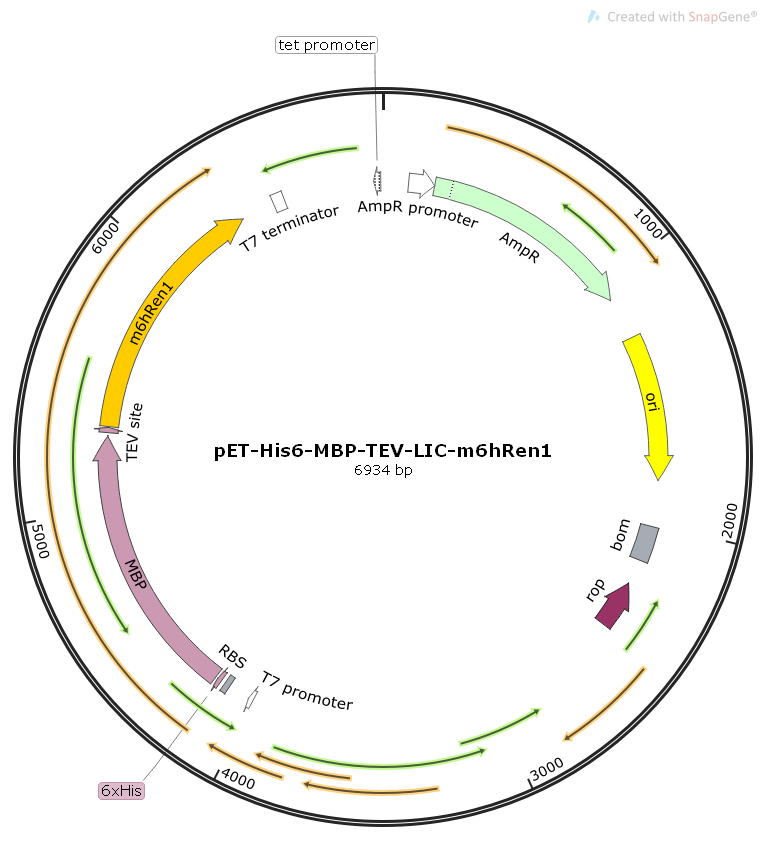


**S9 Fig.** **The full plasmid map for the construct pET-His6-MBP-TEV-LIC-m6hRen1.**


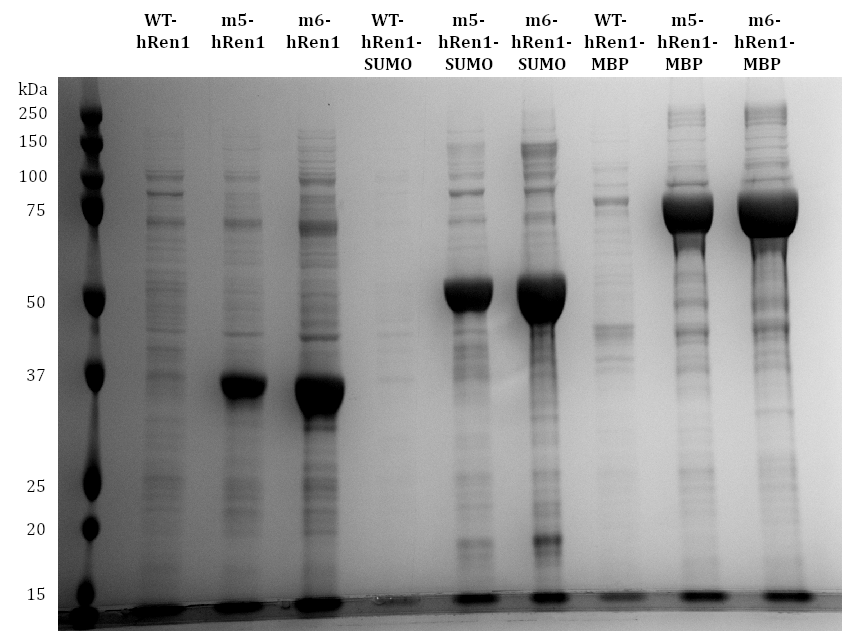


**S10 Fig. SDS-PAGE of the soluble fractions of each of the nine protein variants.** Each sample was concentrated to a factor of 10X relative to the culture medium but normalized by volume to preserve the relative concentrations of protein among variants.

| 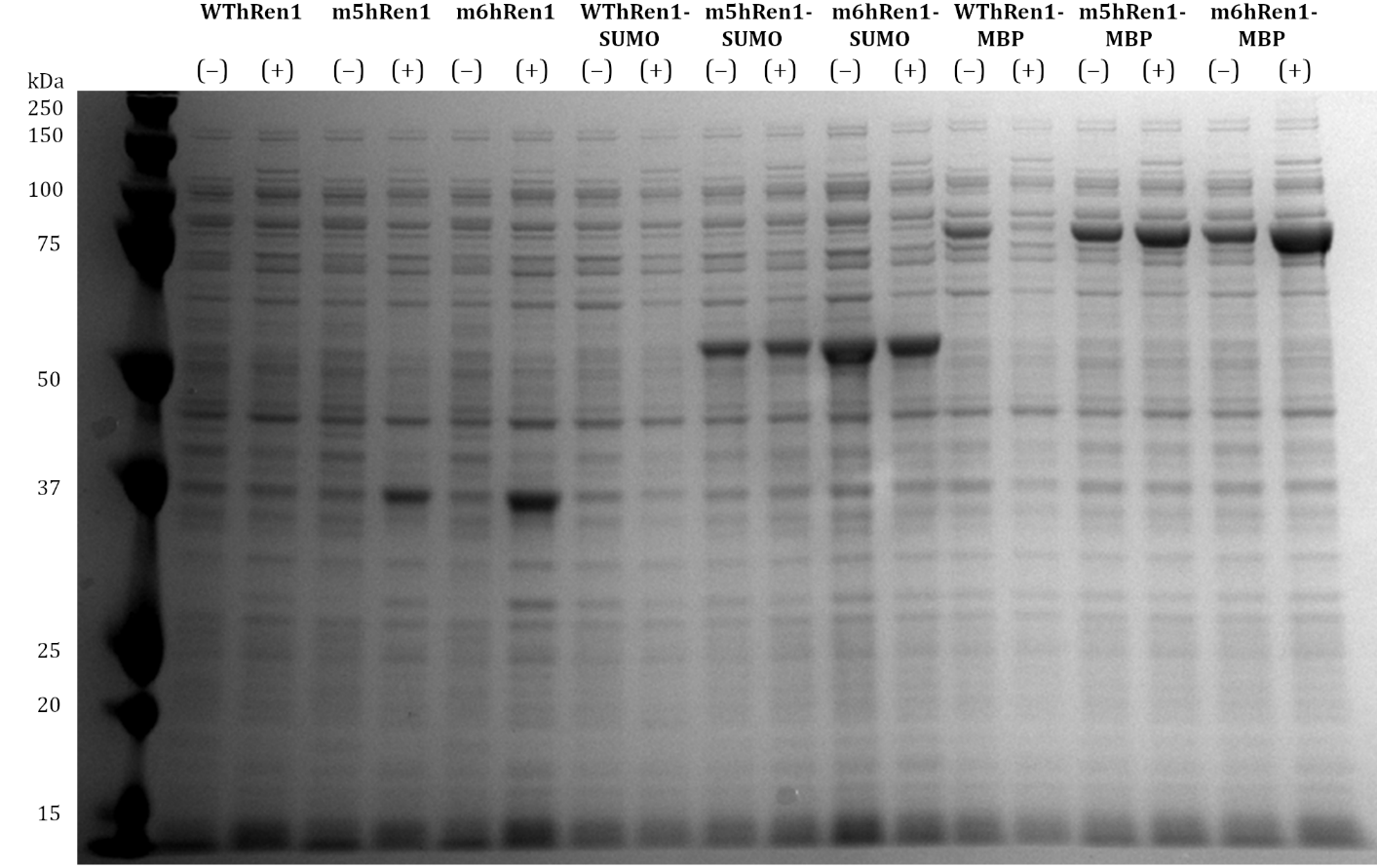 |
| --- |
|  |

**S11 Fig.** **SDS-PAGE of the soluble fraction of the cell lysate**. Batches that had been induced with IPTG are denoted by (+), whereas batches that were not induced with IPTG are denoted by (−).

| 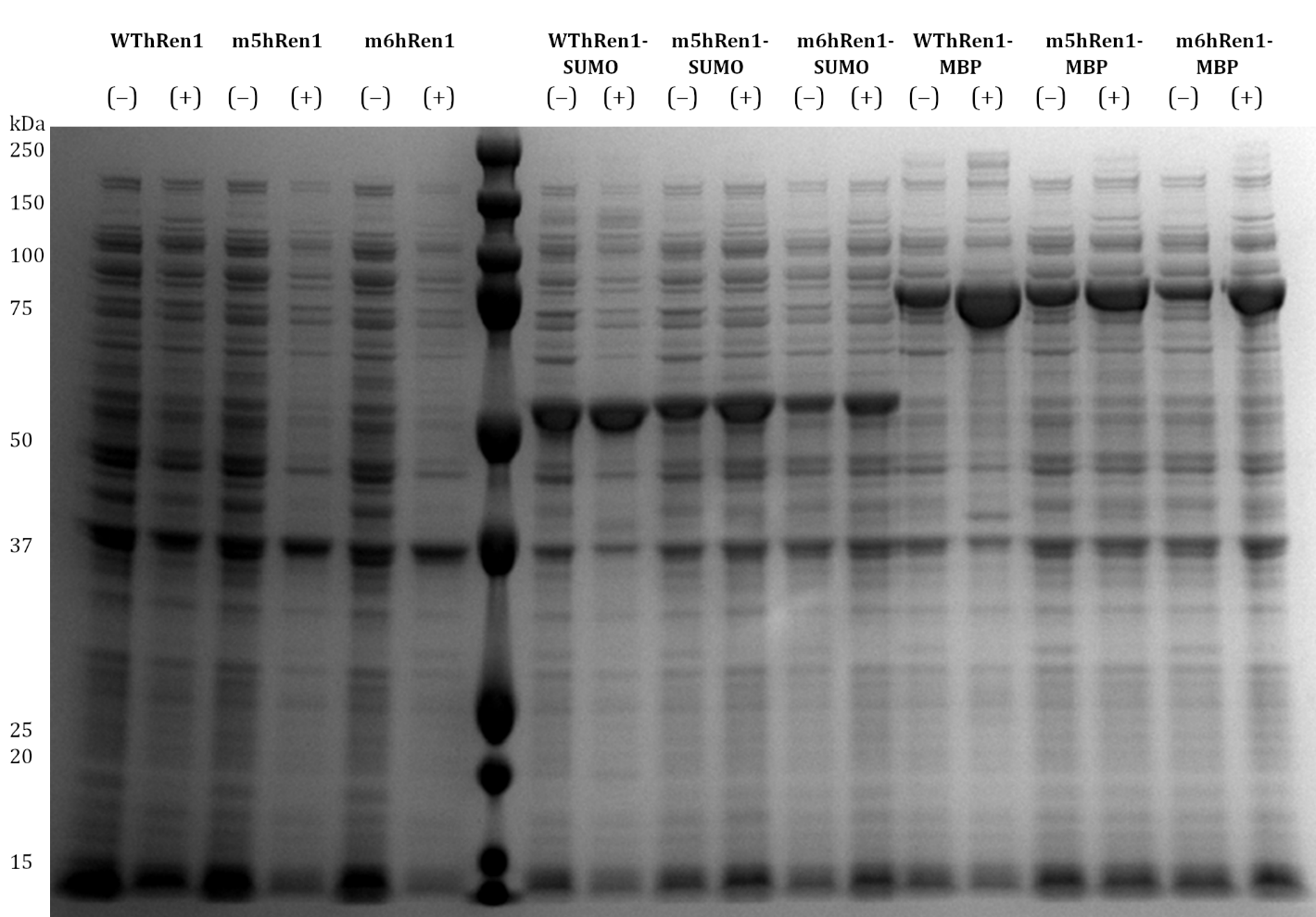 |
| --- |

**S12 Fig.** **SDS-PAGE of the insoluble fraction of the cell lysate**. Batches that had been induced with IPTG are denoted by (+), whereas batches that were not induced with IPTG are denoted by (−).


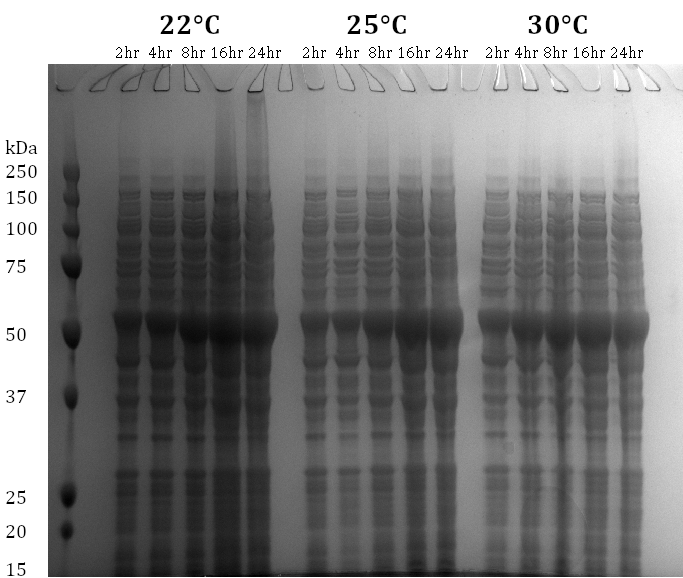


**S13 Fig.** **m6hRen1-SUMO was grown at 22°C, 25°C, and 30°C for up to 24 hours of induction length.** There is increasing amounts of soluble protein expression with diminishing returns beyond 16 hours.

The specific activity of all nine enzyme variants was assessed by holding the concentration of substrate constant and varying the amount of enzyme in each reaction. Specific activity was assessed by measuring the rate of change of optical density at 420nm (mOD_420_) as a function of the amount of enzyme present in the reaction in replicate. The slope of the line resulting from plotting the average values of mOD_420_ min^-1^ gives the specific activity of each enzyme, and is shown in S13-S21 Figs.

**S14 Fig. Specific activity of WThRen1.**

**S15 Fig.** **Specific activity of m5hRen1.**

**S16 Fig. Specific activity of m6hRen1.**

**S17 Fig.** **Specific activity of WThRen1-SUMO.**

**S18 Fig.** **Specific activity of m5hRen1-SUMO.**

**S19 Fig.** **Specific activity of m6hRen1-SUMO.**

**S20 Fig.** **Specific activity of WThRen1-MBP.** Note that the expression level of WThRen1-MBP is so low as to not provide enough active enzyme for reliable analysis.

**S21 Fig. Specific activity of m5hRen1-MBP.**

**S22 Fig.** **Specific activity of m6hRen1-MBP.**

**S23 Fig. m5hRen1 was immobilized on Ni-NTA and ΔmOD420 was used to calculate the velocity of the immobilized enzyme.** The red line is the linear best-fit for the data. These data are scaled to display the small error bars.
