# Supplementary material for: Improved soluble expression and use of recombinant human renalase
Source: PLoS One. 2020 Nov 12;15(11):e0242109. doi: 10.1371/journal.pone.0242109 (PMC7660482; doi:10.1371/journal.pone.0242109)
Supplement: S2 File — (PDF) [file pone.0242109.s002.pdf]

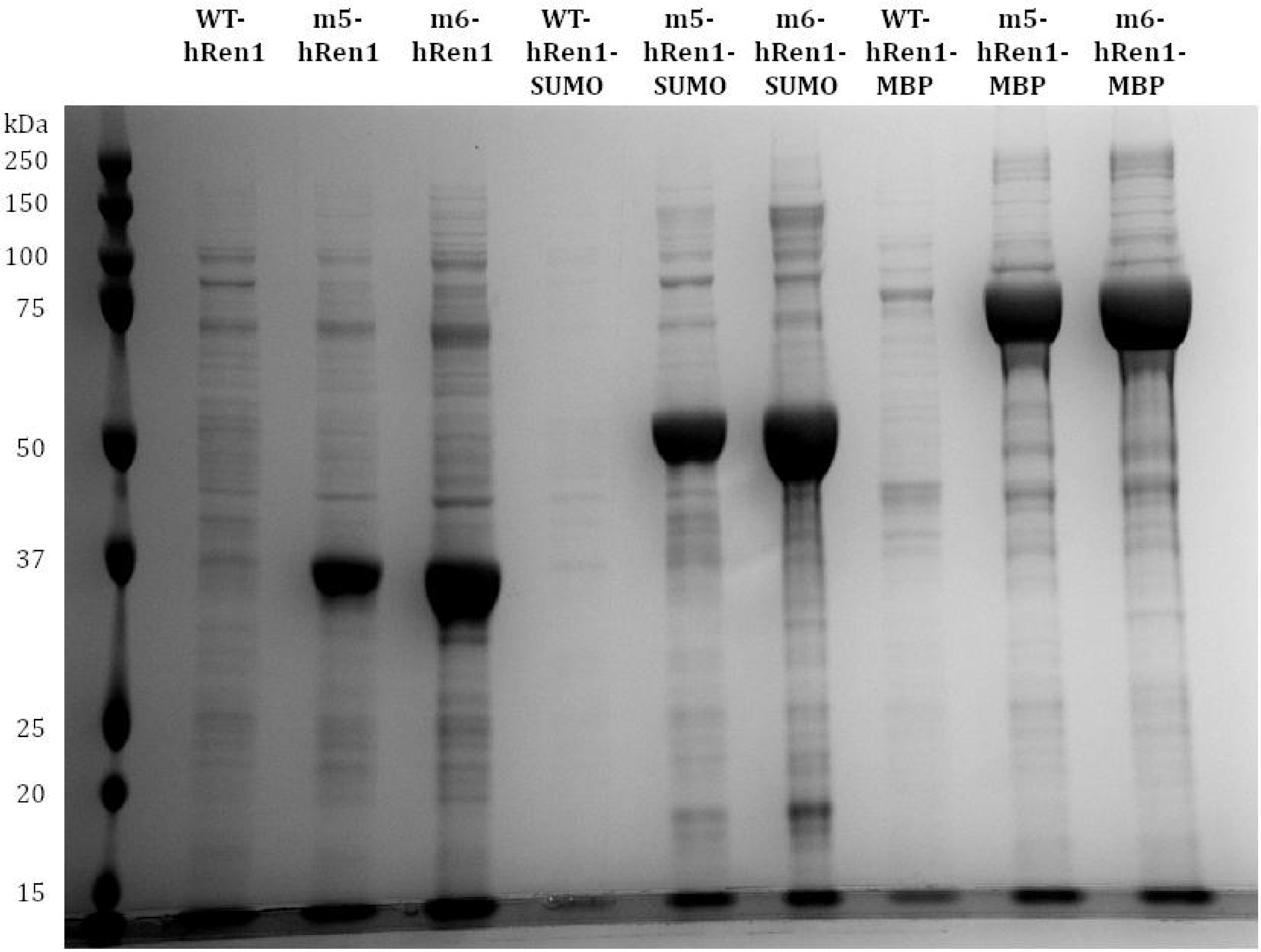
WT-hRen1
m5-hRen1
m6-hRen1
WT-hRen1-SUMO
m5-hRen1-SUMO
m6-hRen1-SUMO
WT-hRen1-MBP
m5-hRen1-MBP
m6-hRen1-MBP
kDa
250
150
100
75
50
37
25
20
15

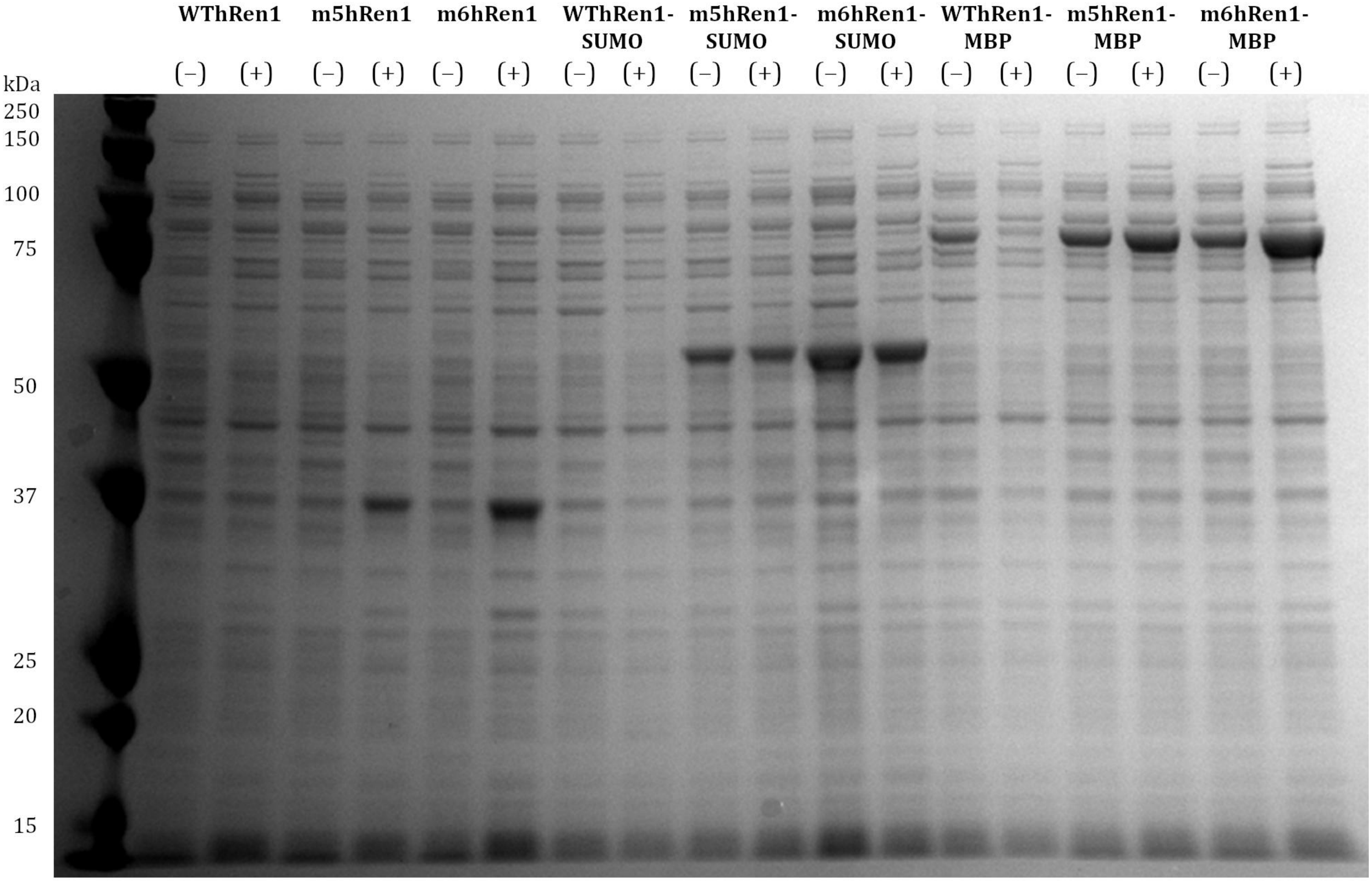

WThRen1
m5hRen1
m6hRen1
WThRen1-SUMO
m5hRen1-SUMO
m6hRen1-SUMO
WThRen1-MBP
m5hRen1-MBP
m6hRen1-MBP
(−) (+) (−) (+) (−) (+) (−) (+) (−) (+) (−) (+) (−) (+) (−) (+) (−) (+)
kDa
250
150
100
75
50
37
25
20
15

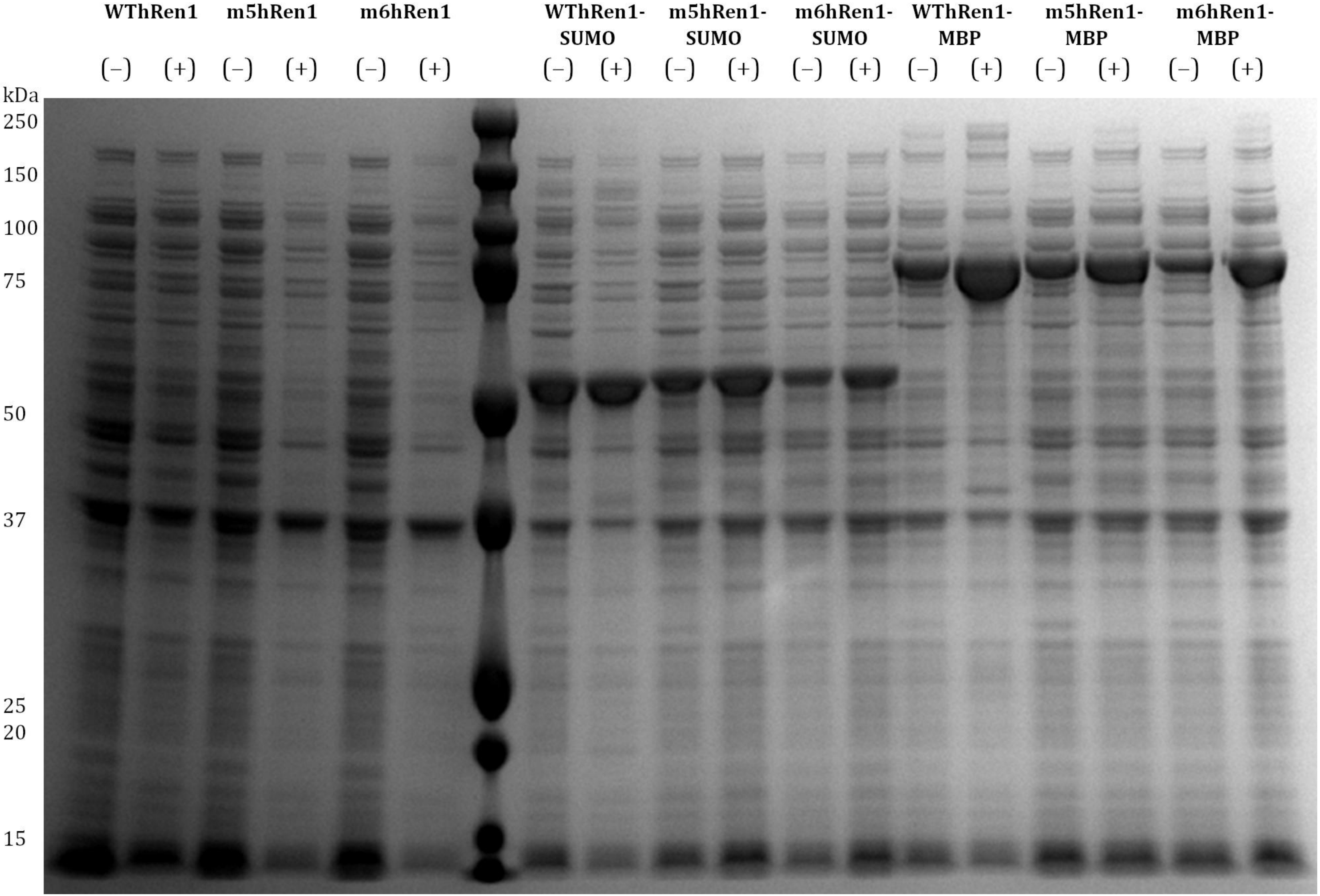
WThRen1
m5hRen1
m6hRen1
WThRen1-SUMO
m5hRen1-SUMO
m6hRen1-SUMO
WThRen1-MBP
m5hRen1-MBP
m6hRen1-MBP
(−) (+) (−) (+) (−) (+) (−) (+) (−) (+) (−) (+) (−) (+) (−) (+) (−) (+)
kDa
250
150
100
75
50
37
25
20
15

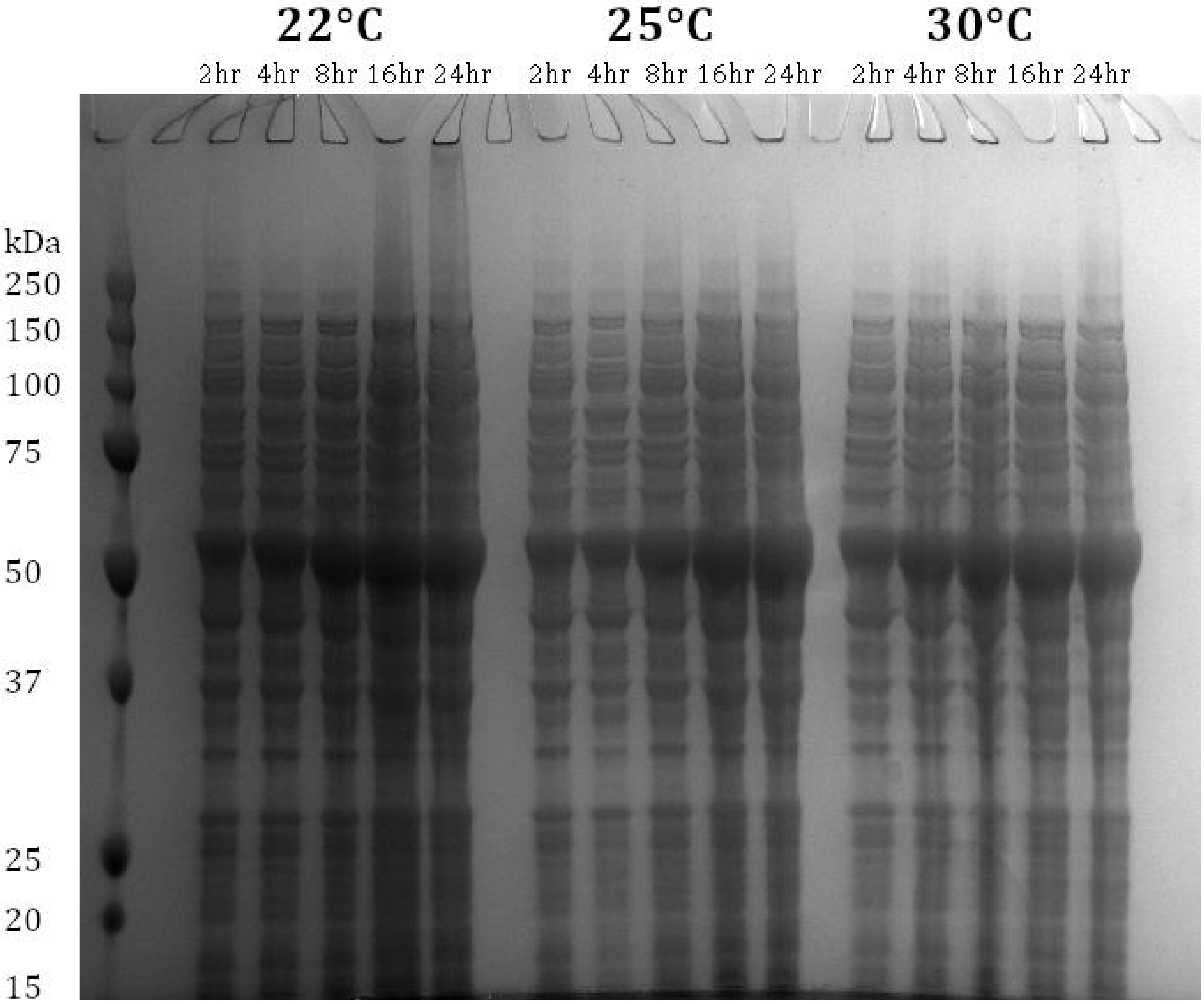
22°C
25°C
30°C
2hr 4hr 8hr 16hr 24hr
2hr 4hr 8hr 16hr 24hr
2hr 4hr 8hr 16hr 24hr
kDa
250
150
100
75
50
37
25
20
15
